# Uncoupling the Vicious Cycle of Mechanical Stress and Inflammation in Calcific Aortic Valve Disease

**DOI:** 10.3389/fcvm.2022.783543

**Published:** 2022-03-09

**Authors:** Nalin H. Dayawansa, Sara Baratchi, Karlheinz Peter

**Affiliations:** ^1^Baker Heart and Diabetes Institute, Melbourne, VIC, Australia; ^2^Department of Cardiology, Alfred Hospital, Melbourne, VIC, Australia; ^3^Department of Medicine, Monash University, Melbourne, VIC, Australia; ^4^School of Health and Biomedical Sciences, RMIT University, Melbourne, VIC, Australia; ^5^Department of Cardiometabolic Health, The University of Melbourne, Melbourne, VIC, Australia

**Keywords:** aortic stenosis (AS), shear stress, calcific aortic valve disease (CAVD), valvular interstitial cells (VIC), mechanotransduction, inflammation, monocytes, platelets

## Abstract

Calcific aortic valve disease (CAVD) is a common acquired valvulopathy, which carries a high burden of mortality. Chronic inflammation has been postulated as the predominant pathophysiological process underlying CAVD. So far, no effective medical therapies exist to halt the progression of CAVD. This review aims to outline the known pathways of inflammation and calcification in CAVD, focussing on the critical roles of mechanical stress and mechanosensing in the perpetuation of valvular inflammation. Following initiation of valvular inflammation, dysregulation of proinflammatory and osteoregulatory signalling pathways stimulates endothelial-mesenchymal transition of valvular endothelial cells (VECs) and differentiation of valvular interstitial cells (VICs) into active myofibroblastic and osteoblastic phenotypes, which in turn mediate valvular extracellular matrix remodelling and calcification. Mechanosensitive signalling pathways convert mechanical forces experienced by valve leaflets and circulating cells into biochemical signals and may provide the positive feedback loop that promotes acceleration of disease progression in the advanced stages of CAVD. Mechanosensing is implicated in multiple aspects of CAVD pathophysiology. The mechanosensitive RhoA/ROCK and YAP/TAZ systems are implicated in aortic valve leaflet mineralisation in response to increased substrate stiffness. Exposure of aortic valve leaflets, endothelial cells and platelets to high shear stress results in increased expression of mediators of VIC differentiation. Upregulation of the Piezo1 mechanoreceptor has been demonstrated to promote inflammation in CAVD, which normalises following transcatheter valve replacement. Genetic variants and inhibition of Notch signalling accentuate VIC responses to altered mechanical stresses. The study of mechanosensing pathways has revealed promising insights into the mechanisms that perpetuate inflammation and calcification in CAVD. Mechanotransduction of altered mechanical stresses may provide the sought-after coupling link that drives a vicious cycle of chronic inflammation in CAVD. Mechanosensing pathways may yield promising targets for therapeutic interventions and prognostic biomarkers with the potential to improve the management of CAVD.

## The Substantial Health Burden of Calcific Aortic Valve Disease

Calcific aortic valve disease (CAVD) is a common cause of mortality and morbidity, with severe aortic stenosis (AS) affecting between 2.9 and 3.4% of elderly patients in the developed world (1, 2). Without treatment, symptomatic, severe AS results in rapid deterioration and death with approximately 50% mortality within 2 years of diagnosis (3). The only treatments effective at preventing heart failure and death in CAVD, however, remain surgical and transcatheter valve replacement, as no effective disease-modifying medical therapies have been developed to date (4). Despite substantial advances in surgical and transcatheter valve replacement (5), severe AS continues to carry a poor prognosis in the modern era, with a three-fold increased risk of mortality at 5 years (6).

Calcific aortic valve disease has previously been described as a “degenerative” valve disease and attributed to mechanical degradation of the valve leaflets over innumerable cardiac cycles as the body ages. Development of CAVD, however, is not ubiquitous in the elderly, and contemporary evidence has established a cycle of chronic inflammation as the driver of progressive aortic valve sclerosis and calcification, highlighting a potential target for therapies to arrest or slow CAVD in the earlier stages (7, 8).

**FIGURE 1:**
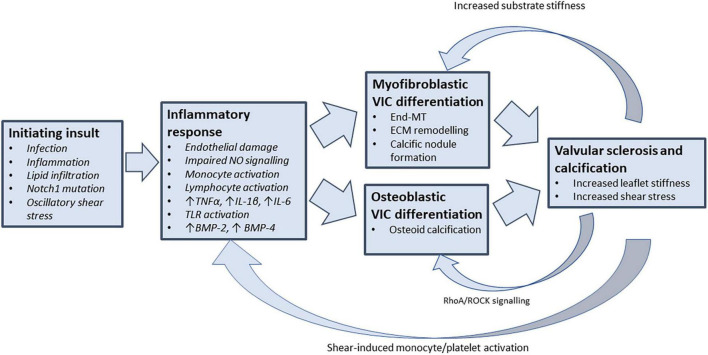
Feedback mechanisms that perpetuate valvular inflammation in CAVD. Understanding the drivers of chronic valvular inflammation are key to arresting the progression of CAVD. Following the initiation of valvular inflammation and stenosis, mechanosensitive feedback pathways may provide the coupling link that promotes further VIC differentiation and valvular inflammation (TNF-α, tumour necrosis factor alpha; IL-1β, interleukin 1-beta; IL-6, interleukin 6; BMP, bone morphogenetic protein; VIC, valvular interstitial cell; End-MT, endothelial-mesenchymal transition; ECM, extracellular matrix; ROCK, rho-associated protein kinase).

Surveillance of patients with CAVD has shown that the rate of stenosis severity progression accelerates as the disease advances; with more rapid rises in mean pressure gradient and aortic valve calcification noted in patients with more severe baseline stenosis (6, 9, 10). This acceleration phenomenon supports the existence of a positive feedback mechanism by which mechanical stresses associated with established AS promote progressive valvular inflammation, sclerosis, and calcification. Mechanotransduction, the process by which mechanical forces are converted into biochemical signals, has emerged as a potential feedback mechanism by which the mechanical stresses of progressive aortic stenosis may promote further valvular inflammation (Figure 1).

This review aims to outline the pathways involved in the initiation and propagation of chronic inflammation of aortic stenosis, with focus on the mechanical stresses experienced by the valve and the mechanotransduction pathways that may act as the perpetuating link in the vicious cycle of chronic inflammation and stenosis.

## Normal Anatomy and Physiology of the Aortic Valve

The aortic valve has unique structural properties to allow it to open with low impedance to unidirectional forward flow and close with sufficient strength to withstand systemic blood pressure loading, all over innumerable cardiac cycles. Each leaflet of the aortic valve comprises three tissue layers with distinct properties arising from the mechanical stresses exerted on the valve leaflets (11). The lamina ventricularis lines the left ventricular side of the leaflet and experiences predominantly laminar steady shear stress from forward blood flow during systole. The ventricularis comprises radially aligned collagen and elastin fibres to maintain elasticity and recoil. During diastole the aortic valve is subjected to compressive stress from pressure loading and tensile stress from radial leaflet lengthening (12). Subsequently, the lamina fibrosa on the aortic side of the valve leaflet is composed of densely packed collagen bundles arranged in a circumferential pattern to maintain structural integrity and transfer pressure load to the aortic root. The intervening middle spongiosa layer comprises predominantly glycosaminoglycans and interstitial cells, with a lower density of collagen fibres interconnecting between the ventricularis and spongiosa layers (13). The spongiosa has been thought to provide lubrication between the two outermost layers, but *in vitro* mechanical simulation using porcine aortic valve leaflets suggest the three layers in fact, move in tandem (14). Valve leaflets are avascular and receive oxygen and nutrients via passive diffusion from the circulating blood pool.

The aortic valve leaflet surface is covered by a layer of valvular endothelial cells (VECs) which is continuous with the endothelial layers of the aorta and the left ventricular endocardium. VECs on both the aortic and ventricular surfaces of the aortic valve are oriented perpendicularly to blood flow in a circumferential fashion, in contrast to vascular endothelial cells which are oriented parallel to the direction of blood flow (15, 16). Haemodynamic forces acting on the healthy valve vary from systole to diastole and have been described in detail in many excellent reviews (17–19). One of the main types of haemodynamic force is shear stress, which is frictional force derived from the movement of blood through the stationary vessel wall and is experienced by the ventricular surface of the leaflets during the systole and the aortic surface as a result of blood pools into the sinuses during diastole.

While the VECs of the ventricular surface are exposed to linear high shear stress from forward blood flow, the aortic surface is exposed to flow vortices within the sinuses of Valsalva and experiences oscillatory low shear stress with directional reversal (17, 20). Similar to the vasculature, regions of the valve that are exposed to oscillatory low shear stress are more prone to calcification and plaque formation (21). One of the potential reasons for the development of an atheroprone phenotype in regions that are exposed to oscillatory low shear stress is the finding that low shear stress upregulates the expression of proatherogenic genes and matrix metalloproteinase activity in endothelial cells (22).

Within the valve matrix itself, the valve is sparsely populated with valvular interstitial cells (VICs), pluripotent cells of mesenchymal origin, which express varying phenotypes under different conditions (23). VICs may differentiate into myofibroblast, chondrocyte, osteoblast, or adipocyte phenotypes and have a key role in development of calcific aortic valve disease (24, 25). In the healthy valve, VICs are involved in regulation and repair of the extracellular matrix (26).

## Inflammation in Aortic Stenosis Pathophysiology: The Initiation-Perpetuation Hypothesis

Calcific aortic valve disease was traditionally thought of as the end-result of passive “wear-and-tear” of the aortic valve over time, an observation supported by the increased prevalence of CAVD with advanced age. The “wear-and-tear” hypothesis, however, did not explain why significant AS only occurs in a fraction of older patients, rather than uniformly developing with advancing age. Established consensus now favours a “response to injury” hypothesis, where progression to haemodynamically significant AS requires three elements: an initiating insult, a subsequent inflammatory response and perpetuating factors, which maintain chronic inflammation and fibrosis (27).

Landmark histopathological studies first characterised the “early lesion” of CAVD; comprising disruption of the endothelium and basement membrane on the aortic side of the leaflet with subendothelial accumulation of lipids, macrophages and T-cells, favouring the leaflet bases over the tips (28). Subendothelial lipid deposition and inflammatory cellular infiltration is observed on both sides of the valve leaflets, with immunohistochemical studies showing increased populations of CD68+ macrophages and CD3+ T cells (28). These findings contrasted with histological observations in healthy ageing of non-specific leaflet tip thickening, reduced spongiosa thickness, and adipose cell interposition between the fibrosa and ventricularis (28). Observations of inflammatory infiltrates and neoangiogenesis in sclerotic valves supported an active chronic inflammation process being responsible for progression of CAVD (29).

There are multiple possible “initiating insults” that have been implicated in CAVD onset, reinforcing the likely heterogenous pathophysiological pathways that lead to CAVD in different patient groups. Injuries to valvular endothelium posed by transient infection or ionising radiation have been shown to initiate valvular inflammation in select patient groups. Tissue polymerase chain reaction (PCR) of excised stenotic aortic valve tissue has shown evidence of previous *Chlamydia pneumoniae* infection in a high proportion of patients (30). Circulating *C. pneumoniae* IgG antibodies and immune complexes have also been associated with an increased risk of CAVD (31). Ionising radiation has been shown to be capable of precipitating aortic valve disease, with a small autopsy series finding valvular endothelial thickening in 81% of young patients who had undergone mediastinal radiation therapy (32). Irradiation has been shown *in vitro* to induce osteogenic differentiation in human aortic VICs, with increased expression of Bone Morphogenetic Protein 2 (BMP2), Runt-related Transcription Factor 2 (RUNX2) and Osteopontin (OPN); all markers of osteogenic VIC differentiation (33).

Following initial injury, lipid deposition appears crucial to propagation of early CAVD, with focal deposition of apolipoprotein (apo) B, apo(a) and apoE identified in all stages of calcific valve lesions; but not in healthy segments of valve leaflet (34). Oxidised lipids trigger further inflammatory response, activating toll-like receptors (TLRs) and activating local macrophages, mast cells and CD4+ and CD8+ T lymphocytes (35, 36). Lipid-lowering therapies, however, have disappointingly shown no effect on the progression of aortic stenosis (37–39).

Mechanical stresses are implicated in both initiation and perpetuation of CAVD. Endothelial disruption, sclerosis and calcification, all predominantly affect the aortic side of the valve at the leaflet bases where it is exposed to turbulent diastolic flow vortices and oscillatory shear stress, more so than the ventricular side which experiences predominantly laminar flow in systole (40). Age is a strong risk factor for CAVD, however, in large scale echocardiographic surveys up to 50% of elderly patients have no significant aortic valve calcification (2). Given CAVD does not occur uniformly with ageing, additional factors may modulate the effect of normal oscillatory shear stress in the initiation of CAVD. As of yet unrecognised genetic or acquired variations may predispose some individuals to aortic valvular inflammation in response to normal oscillatory shear stress.

Mechanical stress likely contributes to the development and progression of stenosis in congenital bicuspid aortic valves (BAV), which are associated with a similar pattern of sclerosis and calcification as CAVD in tricuspid aortic valves, but with earlier onset and more rapid progression (41). BAV results in increased flow vortices in the aortic root and significant increases in wall shear stress (WSS) along the ascending aorta and oscillatory shear index (OSI) on the aortic side of the valve (42, 43). This abnormal blood flow in patients with BAV is consistent with asymmetric aortic dilation and aortic valve stenosis. Although the field of valvular mechanobiology is still at its infancy, development of sophisticated *in vitro* models capable of exposing valvular tissue to different haemodynamic forces, has demonstrated that shear stress overload on the BAV ascending aortas leads to aortic medial degradation, endothelial activation, extracellular matrix degradation and bone matrix synthesis (44–46). In both tricuspid and bicuspid valves, areas of maximal calcification have been shown to correspond to areas of maximal mechanical stress on the aortic side of the valve (47).

## Inflammatory Response to Injury in Calcific Aortic Valve Disease

The nature of calcific aortic valve disease as a chronic inflammatory disease has been well established over the last three decades (8, 48). Normal heart valve leaflets contain few CD45+ leucocytes, which are predominantly macrophages and dendritic cells (49). Stenotic aortic valves show histological features of neovascularisation and chronic inflammatory infiltrates, with aggregates of mononuclear cells, CD3+ and CD4+ T cells and CD20+ B cells (50, 51). CD68+ monocytes adhere selectively on the aortic side of the valve leaflet at areas of higher shear stress (52).

Circulating inflammatory markers are raised in aortic stenosis, with peripheral flow cytometry showing significantly increased levels of circulating intermediate-phenotype monocytes in patients with aortic stenosis compared with matched control patients (53). Elevated levels of C-reactive protein (CRP) have been demonstrated in CAVD but have not been shown to correlate with disease severity or progression in larger observational trials (54–56). A sub-study of the SEAS randomised controlled trial of lipid-lowering therapy in aortic stenosis showed a weak correlation between high sensitivity CRP rise and progression of AS (57).

Increased shear stress caused by aortic stenosis results in unfolding of large von Willebrand factor (VWF) multimers and subsequent degradation by the ADAMTS13 metalloproteinase. This results in reduced circulating levels of large VWF multimers with consequent impaired platelet adhesion/aggregation in patients with severe AS, which normalises after valve replacement surgery (58). The syndrome of aortic stenosis in combination with gastric angiodysplasia, often associated with bleeding, is collectively termed Heyde’s syndrome (59).

Macrophage infiltration is observed in both early and advanced lesions of CAVD (28, 60). Macrophages participate in tissue inflammation as one of two polarised phenotypes. Classically activated M1 macrophages are induced by pro-inflammatory stimuli such as interferon gamma (IFNγ), tumour necrosis factor alpha (TNFα) or lipopolysaccharide exposure; and combat pathogens and tumour cells via the production of reactive oxygen species and inducing Th1 responses via interleukins IL-12 and IL-23 production (61). M2 macrophages are induced by IL-4 and IL-13 and mediate tissue healing and repair in the absence of active pathogens. Calcified human aortic valve leaflets show increased levels of macrophage infiltration, with an increase in CD11c+ M1 macrophages and a reduction in CD206+ M2 macrophages compared with non-calcified aortic valve leaflets (62). Tissue macrophages alter the local valvular microenvironment by releasing of TNFα and transforming growth factor beta (TGFβ), promoting myofibroblastic differentiation of VICs and calcification (63, 64).

Neutrophils have been implicated in calcific cardiovascular diseases, and increased neutrophil-lymphocyte ratio (NLR) has been shown to correlate with coronary artery calcium scores and severity of aortic stenosis, with normalisation of NLR after TAVR (65–67). The role neutrophils play in the development of calcific cardiovascular disease, however, remains unclear and may relate to activation of platelets via neutrophil extracellular traps (NETs) (68). Increased neutrophil activation has been demonstrated in severe AS with NET presence in aortic valve leaflets and elevated plasma citrullinated histone H3 (69).

## The Role of Valvular Endothelial Cells in Development of Calcific Aortic Valve Disease

Valvular endothelial cells (VECs) form the interface between the aortic valve leaflets and the surrounding microenvironment. Focal disruption of the fibrosa endothelium with subendothelial apolipoprotein deposition are observed in histological studies of human aortic valves with early CAVD, implicating VECs in the initiation of CAVD (28, 34). Endothelial cells of diseased aortic valves show increased expression of cell adhesion molecules such as ICAM1, VCAM1, and E-selectin which may recruit inflammatory cells to the valve leaflet (70, 71).

Derangements in endothelial redox signalling also play a role in the development of CAVD. In patients with aortic stenosis, markers of oxidative protein damage increase in correlation with rising mean pressure gradients (72). Nitric oxide (NO) production via endothelial nitric oxide synthase-3 (NOS3) is critical to cardiac valve development, with *Nos3* knockout resulting in a bicuspid valve phenotype in mice (73). Endothelial-derived NO inhibits calcification of porcine aortic VICs *in vitro* and modulates Notch1 signalling (74). Post-translational modification of the deubiquitinase USP9X has been demonstrated as a mechanism for NO-mediated inhibition of myofibroblastic porcine aortic VIC differentiation (75). In models of vascular calcification, endothelial-derived NO inhibits TGFβ signalling in vascular smooth muscle cells, which may be an additional mechanism by which NO signalling inhibits pathological VIC differentiation (76). Disruption of protective redox signalling hence may be a mechanism by which endothelial cell damage promotes early CAVD.

Under the influence of stimuli such as TGFβ and BMPs, VECs may express mesenchymal cell-type characteristics in a process known as endothelial to mesenchymal transition (End-MT). The process of End-MT is crucial to the embryological development of cardiac structures such as the atrioventricular fibrous continuity, valvular apparatus and interventricular septum (77). *In vitro*, End-MT can be identified by the reduction in expression of endothelial genes and proteins such as CD21, VE-cadherin and endothelial NOS; with commensurate increase in expression of mesenchymal genes and proteins such as alpha smooth muscle actin (αSMA), calponin, smooth muscle protein 22α (SM22α) and versican (78). End-MT is proposed to play a function in maintaining aortic valve homoeostasis by replenishing a mesenchymal cell type population in order to regulate extracellular matrix turnover, however, End-MT may also play a pathological role in the development of CAVD (79, 80). Proinflammatory cytokines such as TNFα and IL-6 have been shown to induce End-MT in porcine aortic VECs *in vitro*, and cells displaying evidence of End-MT can be found within the fibrosa of calcified human aortic valve leaflets (80, 81).

## Altered Shear Stress in Calcific Aortic Valve Disease Promotes Local Inflammation

In normal conditions, the advancing column of blood flow through the aortic valve generates a trailing ring of vortices within the sinuses of Valsalva which aids in aortic valve closure at the end of systole (82, 83). These normal conditions expose the ventricular side of the aortic valve leaflets to steady shear conditions, while the aortic surface is exposed to low magnitude oscillatory shear stress (40). As CAVD progresses, the shear stresses experienced by both sides of the aortic valve leaflets are altered. Reduction of the effective valve orifice area in aortic stenosis results in increased velocity of transaortic blood flow and increased high magnitude steady shear stress on the ventricular side of the aortic valve leaflets (40). As blood flow velocity increases, there is greater turbulence within the aortic root and sinuses of Valsalva, imparting increased magnitude of oscillatory shear stress and turbulent flow profiles upon the ventricularis (84).

Under steady shear conditions normally experienced by the ventricularis, cultured porcine aortic VECs express an anti-inflammatory and anti-oxidative gene expression phenotype manner similar to but distinct from that of vascular endothelial cells (85). Compared to high magnitude steady shear stress, low magnitude steady shear and both low and high magnitude oscillatory shear promotes End-MT in cultured porcine aortic VECs as evidenced by increased αSMA expression and decreased expression of the endothelial cell adhesion protein PECAM1 (86).

Exposure of porcine aortic valve leaflets to supra-physiological oscillatory shear stress magnitudes results in increased expression of BMP4, TGFβ1, and the matrix metalloproteinases MMP2 and MMP9 in the fibrosa; all key mediators of VIC differentiation and extracellular matrix (ECM) remodelling (87). Alterations in shear stress frequency did not elicit the same effect. BMP4 expression in endothelial cells in response to oscillatory shear stress additionally has been linked to stimulation of monocyte adhesion and generation of reactive oxygen species (ROS) (88, 89). Upregulation of the transcription factor Snail has been shown to be essential for End-MT to occur in response to low magnitude oscillatory shear in vascular endothelial cells, however, this mechanism linking altered shear stress to End-MT has not been confirmed in valvular endothelium (90).

Increased shear stress has been linked to increased oxidative stress and inflammatory cell recruitment in models of vascular atherogenesis. Increased magnitude steady shear stress induces ROS production and inducible NO synthase (iNOS) activity in aortic vascular endothelial cells *in vitro* (91, 92). In human aortic valves, areas of calcification show increased oxidative stress markers, and reduced antioxidant enzyme expression co-localised with increased VCAM1 and CD31 expression, suggesting a link between oxidative stress and inflammatory activation (93, 94). Exposing human aortic endothelial cells to oscillatory shear stress results in ROS generation via BMP4 production and promotes increased monocyte adhesion (88).

**FIGURE 2:**
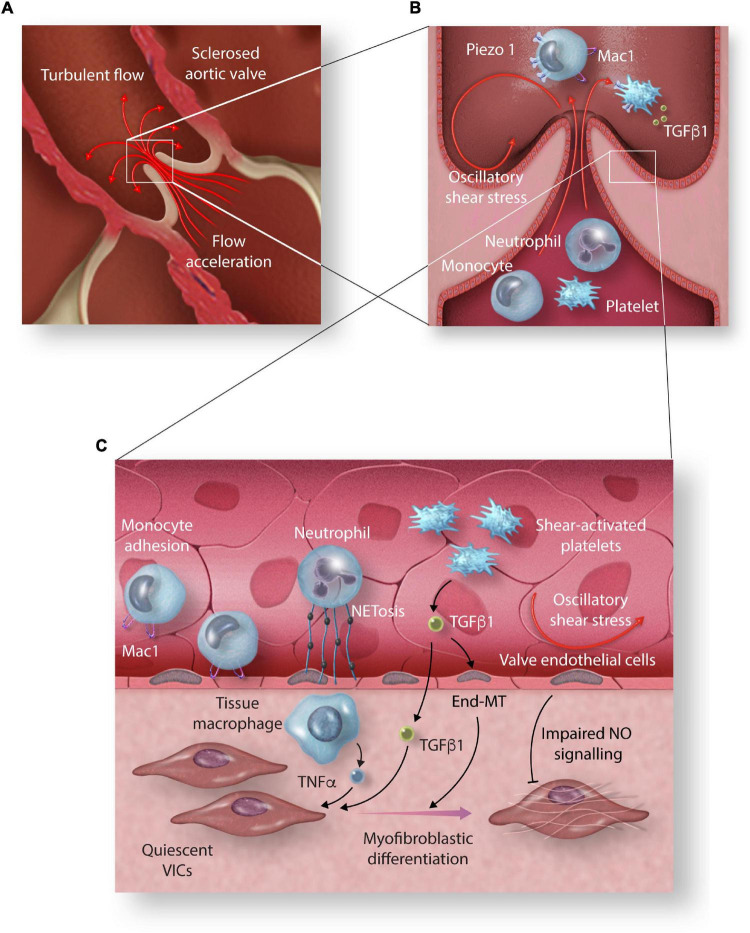
The effect of high shear stress on circulating cells and their role in promoting valvular inflammation. **(A)** Acceleration of blood velocity through the narrowed aortic valve exposes circulating cells to high shear stress (HSS) and creates turbulent flow vortices on the aortic side of the aortic valve leaflets. **(B)** HSS induces increased expression of monocyte adhesion molecule Mac1 mediated by increased expression and activation of Piezo1. **(C)** TNF α released by tissue macrophages, TGFβ released by HSS-activated platelets, and NETosis of shear activated neutrophils promote myofibroblastic differentiation of aortic valve interstitial cells (VICs). Increased magnitude oscillatory shear stress (OSS) promotes End-MT and impaired protective NO signalling (End-MT, endothelial-mesenchymal transition; Mac1, macrophage-1 antigen; TGF-β1, transforming growth factor β1; TNF-α, tumour necrosis factor-alpha; NET, neutrophil extracellular trap; NO, nitric oxide).

Altered shear stresses also contribute to activation and recruitment of circulating cells as they pass through the stenotic aortic valve, which may in turn promote VIC differentiation and local valvular inflammation (Figure 2). The role of oscillatory and low magnitude steady shear stress states in recruiting monocytes has been well established in atherosclerosis disease models. Subjecting monocytes to oscillatory shear stress or complex flow reversals *in vitro* results in upregulation of cellular adhesion molecules and increased binding of monocytes to endothelial cells (95, 96).

Exposure of healthy platelets to high laminar shear rates in aortic stenosis results in increased platelet activation as measured by glycoprotein IIb/IIIa activation and P-selectin surface expression (97–99). There is also evidence of platelet dysfunction in early stages of CAVD, with platelet nitric oxide (NO) resistance and increased platelet aggregability demonstrated in moderate aortic stenosis (100). High shear conditions promote increased release and activation of platelet-derived TGFβ1 both *in vitro* and in mouse models of aortic stenosis (101, 102). Progression of AS in murine models is attenuated by the knockout of platelet TGFβ1, supporting a key role of shear-induced TGFβ1 release and of platelets in general in the pathogenesis of aortic stenosis (103).

Microparticles are small membrane vesicles released by ectocytosis, budding of the plasma membrane, from leucocytes, endothelial cells or platelets (104). Microparticles (MPs) express parent cell surface molecules that may affect cell-cell interactions and have been implicated in atherosclerosis, thrombosis and inflammatory disorders (105, 106). Increased levels of platelet-derived microparticles (PMPs), endothelial-derived microparticles (EMPs) and leucocyte-derived microparticles (LMPs) have been observed in patients with severe aortic stenosis and correlate with increased monocyte activation (107). Subjecting whole blood to increase *in vitro* shear conditions results in linear increase in PMP production, supporting transvalvular shear stress as a primary driver of MP release in patients with aortic stenosis (107).

## Side-Specific Pro-Calcific Characteristics of the Lamina Fibrosa

Histological descriptions of CAVD have long identified that the early disease process predominantly affects the aortic side of the leaflets rather than the ventricularis (28). When the high magnitude steady shear conditions experienced by the ventricularis and the low magnitude oscillatory shear conditions experience by the fibrosa are applied to cultured human umbilical vein endothelial cells (HUVECs) *in vitro*, the steady shear conditions induce a protective response with reduced expression of monocyte chemoattractant protein-1 (MCP1) and increased expression of krüppel-like transcription factor-2 (KLF2) and nephroblastoma overexpressed protein (NOV) (20). VECs however, have many unique properties compared to other endothelial cells, and further study has suggested that VECs of the fibrosa possess properties predisposing to calcification compared to VECs of the ventricularis. VECs on the fibrosa side of healthy porcine aortic valve leaflets exhibit increased expression of osteoprotegerin (OPG) protein and downregulation of anti-calcific genes such as *OPG* and parathyroid hormone (*PTH*) at baseline (108). Exposing the aortic surface of porcine aortic valve leaflets to abnormal steady shear stress *in vitro* induces increased endothelial expression of TGFβ1 and BMP4 which in turn mediates increased expression of ICAM1 and VCAM1. Exposure of the aortic surface, however, to physiologically normal low-magnitude oscillatory shear stress and exposure of the ventricular surface to either physiologically normal steady shear stress or abnormal oscillatory shear stress does not elicit the same change in TGFβ1 or BMP4 expression (109). The presence of differing side-specific behaviours suggests that not all VECs are equivalent, adding to the complexity of performing and interpreting *in vitro* experiments using VEC cultures.

## The Role of Valvular Interstitial Cells in Calcific Aortic Valve Disease Propagation

**FIGURE 3:**
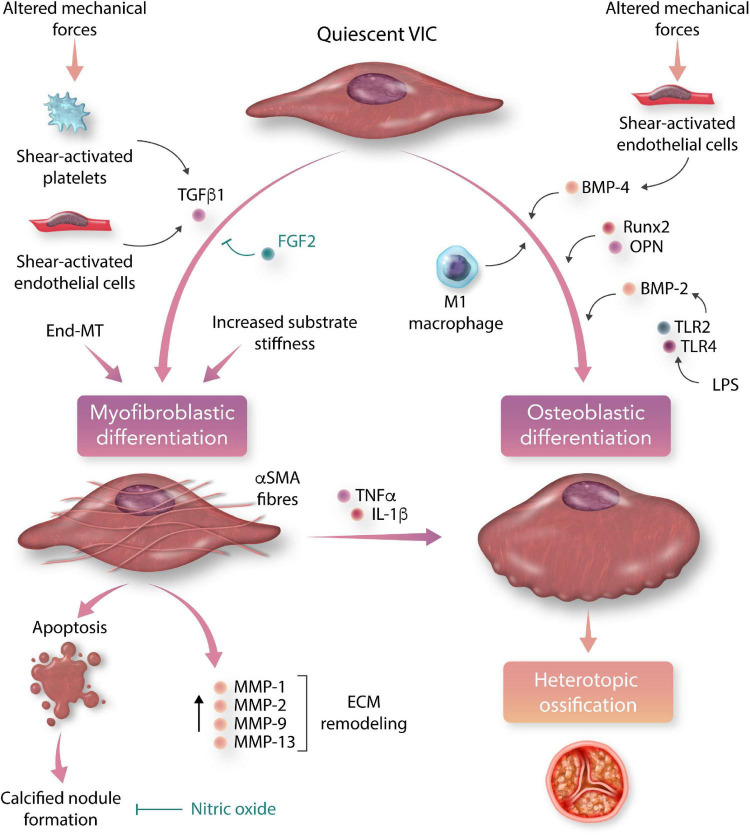
Myofibroblastic and osteoblastic differentiation of aortic valvular interstitial cells (VICs) in calcific aortic valve disease (CAVD). Under the influence of circulating and local chemical stimuli, pluripotent valvular interstitial cells differentiate into myofibroblastic and osteoblastic phenotypes, which are the major cellular effectors of valve leaflet remodelling and calcification (TGFβ1, transforming growth factor beta-1; BMP, bone morphogenetic protein; OPN, osteopontin; TLR, toll-like receptor; LPS, lipopolysaccharide; End-MT, endothelial-to-mesenchymal transition; FGF2, fibroblast growth factor-2; αSMA, alpha-smooth muscle actin; TNFα, tumour necrosis factor-alpha; IL-1β, interleukin 1-beta; MMP, matrix metalloprotein; ECM, extracellular matrix).

All three layers of the aortic valve leaflets are populated with VICs, which are responsible for the regulation and repair of valvular tissue. Under the influence of pro-inflammatory signals and mechanical stress, quiescent VICs differentiate into active phenotypes, which play a major role in the propagation of valvular sclerosis and calcification following an initial insult (Figure 3) (110).

In the presence of TGFβ1, quiescent VICs differentiate into activated VICs with a myofibroblast phenotype, characterised *in vitro* by expression of the contractile protein alpha-smooth muscle actin (αSMA). Application of tensile stress to the collagen matrix has been shown to greatly enhance the myofibroblastic response to TGFβ1 with VICs displaying increased expression of αSMA and increased contractile properties (111). Aortic endothelial cells increase secretion of TGFβ1 in response to the application of increasing steady shear forces (112).

Given the role of TGFβ1 in both End-MT of VECs and myofibroblastic differentiation of VICs, interactions between VEC and VIC may contribute to the development of CAVD. Under physiological steady shear conditions *in vitro*, the presence of co-cultured VECs reduces αSMA expression and appears to protect against Myofibroblastic differentiation of porcine VICs (113). Similarly, co-culture of VICs with ovine VECs protects against End-MT and calcification despite the influence of TGFβ1 (80, 114). These observations suggest that disruption of normal VIC-VEC interactions may precipitate End-MT and myofibroblastic differentiation in the early development of CAVD.

Activated myofibroblast-phenotype VICs secrete the collagenases MMP1, MMP2, MMP9, and MMP13 and appear to play a key role in ECM remodelling in response to injury (115). Myofibroblastic differentiation of porcine VICs can be suppressed by basic fibroblast growth factor (FGF2), which inhibits TGFβ1-mediated Smad transcription factor activation and αSMA expression, with subsequent reduction in valve leaflet fibrosis and nodule formation (116). When exposed to osteogenic medium and TGFβ1, activated ovine and porcine VICs aggregate and undergo apoptosis, forming a nidus for formation of dystrophic calcific nodules typical of advanced CAVD (117, 118). TGFβ1 stimulated calcific nodule formation by porcine aortic VICs can be inhibited conversely by exposure to nitric oxide donors, suggesting a role for oxidative stress in the promotion of valvular calcification (119).

## Cyclic Tensile Stress Promotes Extracellular Matrix Remodelling

Studies of the effect of shear stress on valvular endothelium have flourished, in no small part due to the extensive prior examination of the role of shear stress in vascular atherosclerosis. The aortic valve, however, is also subjected to bending stresses, stretch and pressure loading with each cycle of opening and closure, and less is known about the role these stresses play in the development of CAVD. In diastole, tensile stress is exerted on the convex ventricular side of the leaflet with compressive stress of the concave aortic side. Conversely as the leaflet opens in systole the aortic side becomes convex and experiences tensile stress, and the concave ventricularis experiences compressive stress (120). Throughout the cardiac cycle the leaflets experience net tensile stress, and under normal loading conditions experience radial and circumferential stretch of approximately 10% in diastole (121). Increasing systemic blood pressure increases the magnitude of cyclic stretch, and diastolic hypertension has been associated with poorer outcomes in patients with AS (122, 123).

Applying cyclic mechanical stretch to cultured porcine aortic VICs induced increased type III collagen production and cycle increased ALP expression following addition of osteogenic medium, suggesting that mechanical loading in diastole may promote increased ECM remodelling (124). Application of physiological cyclic stretch to porcine AV leaflets enhances TGFβ1 mediated myofibroblastic differentiation of VICs and increased collagen synthesis in a synergistic fashion (125). Escalating the magnitude of cyclic stretch to supraphysiological levels increases expression of MMP-1, -2, and -9 and increases collagen deposition (121).

The relationship between cyclical loading and AVIC differentiation and ECM remodelling reinforces the sensitivity of the aortic valve microenvironment to mechanical stress. The pattern of increased MMP activity and disorganised collagen deposition induced by cyclic strain is observed in human aortic stenosis, but not in aortic regurgitation (126). Systemic blood pressure is the major determinant of tensile stress in the aortic valve, and the effect of blood pressure lowering on CAVD progression has not been directly studied. The Ramipril in Aortic Stenosis (RIAS) trial examined the potential of ACE inhibitor therapy to reduce LV hypertrophy in AS and showed no significant difference in AS progression at 12 months compared with placebo, however, a significant difference in blood pressure between groups was not achieved either (127).

## Increased Valve Leaflet Stiffness Promotes Progression of Calcific Aortic Valve Disease

Once valvular sclerosis is established, the mechanical properties of valve leaflets directly influence VIC differentiation and classification in a positive feedback loop promoting the progression of CAVD. Culture of naïve VICs in osteogenic medium on stiffer substrate promotes myofibroblastic differentiation and increased αSMA expression (128–130). VICs cultured on a more compliant collagen-based substrate, however, exhibited osteoblastic characteristics, but only when cultured in a calcifying medium (128). ECM stiffness also modulates VIC response to TGFβ1 which induces calcified apoptotic nodule formation on stiff, but not compliant, matrix substrates (128). As formation of αSMA stress fibres increases cellular contractility and tension this may in turn promote further myofibroblastic VIC differentiation in a positive feedback loop (131).

## Osteoregulatory Pathways Implicated in the Development of Calcific Aortic Valve Disease

Valvular calcification is a key step in the progression of advanced CAVD and has been associated with local and systemic derangements in the OPG/RANK/RANKL bone metabolism regulatory pathway. An understanding of the OPG/RANK/RANKL pathway aids in the identification of the downstream pro-calcific pathways that may link mechanical stresses to the development of CAVD.

Receptor-activator of nuclear factor kappa B (RANK) is a cell surface receptor expressed primarily on osteoclasts, macrophages and dendritic cells (132). Receptor-activator of nuclear factor kappa B ligand (RANKL), a membrane protein expressed on the surface of T cells in lymphoid tissue and osteoblasts, binds to the RANK receptor, stimulating osteoclast differentiation and activity and inhibiting osteoclast apoptosis (133).

Osteoprotegerin (OPG) is a member of the TNFα superfamily and acts as a counterregulatory decoy receptor for RANKL, preventing RANK-RANKL binding and hence downregulating osteoclast activity. OPG has a protective effect against arterial calcification, with *Opg* knockout mice exhibiting increased rates of arterial atherosclerosis and calcification (134, 135). Upregulation of RANKL and downregulation of OPG has been observed in calcified human aortic valve leaflets, with RANKL stimulation promoting osteoblastic differentiation of VICs *in vitro* (136). In a hypercholesterolemic mouse model of CAVD, exogenous OPG reduces valvular calcification and functional valvular stenosis (137). The fibrosa layer of the aortic valve displays pro-calcific elements with increased endothelial expression of Bone Morphogenetic Protein-4 (BMP4) and reduced expression of OPG and PTH (108).

The Bone Morphogenetic Protein (BMP) family constitutes several signalling proteins implicated in cartilage and bone formation. BMP-2, -4, -5, -6, -7, and -9 promote cartilage formation and ossification in normal embryonic development; and BMP3 acts as an antagonist, inhibiting BMP2 mediated skeletogenesis (138). Studies of human CAVD have shown increased expression of BMP2 and BMP4 as well as increased downstream pSmad1/5/8 signalling on the fibrosa of calcified aortic valves (89).

Mechanical stress has been shown to induce alterations in the OPG/RANK/RANKL and BMP pathways in other organ systems. Mechanical stress is a major regulator of skeletal bone resorption and deposition, with both tensile and compressive stress promoting osteogenic differentiation of human mesenchymal stem cells *in vitro* (139). Tensile stress applied to human osteoblast-like cells *in vitro* stimulates initial increased expression of BMP2 and BMP4 which returns to baseline with prolonged stretching (140).

## Osteogenic Differentiation of Valvular Interstitial Cells

Under the influence of osteogenic medium, aortic valve VICs differentiate down an osteoblastic pathway and are strong mediators of aortic valve calcification (25). Osteoblastic phenotype VICs are characterised *in vitro* by expression of alkaline phosphatase (ALP), bone sialoprotein and Runt-related Transcription Factor 2 (RUNX2); a marker of terminal osteoblastic differentiation. Osteoblastic differentiation of human aortic VICs *in vitro* is promoted by exposure to BMP2, RUNX2, and osteopontin (110, 141). Stimulation of TLR2 and TLR4 by lipopolysaccharides has also been shown to induce increased expression of BMP2, RUNX2, and ALP in human aortic valve VICs (142). Pro-inflammatory cytokines such as TNFα and IL-1β have also been shown to induce a transition from myofibroblastic to osteogenic phenotype in porcine VICs, with reduced αSMA expression and increased RUNX2 expression (143). Silencing of αSMA in myofibroblastic phenotype VICs reduces transition to osteoblastic differentiation and development of calcification (144). Incubation with conditioned medium from M1-differentiated macrophages has been shown to induce osteoblastic differentiation of VICs in a partially TNFα and IL-6-dependent manner (62).

## Mechanotransduction Pathways as Possible Therapeutic Targets in Calcific Aortic Valve Disease

Mechanotransduction, the conversion of mechanical stress into biochemical signals, is mediated by different groups of mechanoreceptors ranging from ion channels and adhesion molecules to the components of the cytoskeleton and transcription factors (19). Identifying the mechanosensitive pathways which link altered mechanical stresses to inflammation and progression of CAVD may provide novel therapeutic targets.

Endothelial cells detect shear stress by a number of mechanisms including G-protein-coupled receptor activation, tyrosine kinase receptor activation, mechanosensitive ion channel activation and conformational changes in the glycocalyx and primary cilia (145). Shear-mediated activation of cellular adhesion molecules results promotes leucocyte recruitment, and an intracellular signal cascade results in altered protein and gene expression, increased NO production and cytokine production; all of which may contribute to the cellular processes that drive CAVD (95).

Yes-associated protein 1 (YAP) and Transcriptional coactivator with PDZ-binding motif (TAZ) are transcriptional co-regulators of the Hippo growth regulatory pathway and act as key signals in mechanotransduction; the pathways by which mechanical stresses are translated into cellular responses (146). Focal upregulation of cytoplasmic and nuclear YAP is found in calcified portions of stenotic human aortic valve leaflets, and culture of human aortic VICs from stenotic valves on increasingly stiff substrate resulted in increasing nuclear translocation of YAP (147, 148).

The RhoA/ROCK signalling pathway has been shown to be involved in aortic valve leaflet mineralisation in response to cyclic stretch; and ROCK inhibition interrupts osteoblastic differentiation of human aortic VICs on a compliant substrate (149, 150).

Mechanosensitive ion channels trigger ion currents in response to mechanical stresses and are implicated in a diverse range of sensory functions such as touch, hearing and pain. Piezo1 is a mechanosensitive ion channel that generates ion currents in response to increased membrane tension, shear stress, pressure and stretch (151–155). Piezo1 channels regulate embryonic cardiac valve development in response to mechanical stress, with impaired valve formation observed in *piezo1* knockout zebrafish (156). Piezo1-mediated calcium influx has been demonstrated in shear-stress induced monocyte activation, and monocyte populations from patients undergoing transcatheter aortic valve replacement (TAVR) have shown upregulation of Piezo1 (52).

The Notch signalling pathway is a highly conserved signalling pathway responsible for cell proliferation and tissue development. Notch signalling is regulated by mechanical stress and plays a key role in cardiogenesis and maintenance of cardiac tissue homoeostasis (157, 158).

Genotyping of a familial cluster with an autosomal dominant pattern of congenital bicuspid and tricuspid calcific aortic valve disease revealed the loss-of-function *R1108X* mutation encoding a premature stop codon in the Notch1 extracellular domain (73). *Notch1* knockout in mice results in increased expression of *Bmp2*, *Runx2*, and aortic valve calcification, suggesting that normal Notch signalling may protect against pathological osteogenic differentiation of VICs (159, 160). Analysis of explanted valves from adults undergoing aortic valve replacement identified upregulation of long non-coding RNA H19 which reproducibly suppressed *NOTCH1* gene expression, further implicating *NOTCH1* inhibition in sporadic CAVD (161). The micro-RNA miR-34a has also been identified in explanted valves as an inhibitor of Notch1 mRNA expression, and administration of an miR-34a inhibitor results in attenuation of aortic valve calcification and stenosis in a murine wire-injury model of CAVD (162).

The effect of Notch1 inhibition is modulated by mechanical stress, and aortic VICs isolated from *Notch1*^+/–^ genotype mice and exposed to mechanical strain exhibit exaggerated αSMA expression, myofibroblastic differentiation and dystrophic calcific nodule formation compared to wild-type VICs (163). Similarly, exposure of human aortic VICs to oscillatory shear stress (OSS) in combination with a Notch1 inhibitor resulted in increased αSMA expression, where Notch1 inhibition alone resulted in calcific nodule formation with reduced αSMA expression (164). Genetic variations in *NOTCH1* may predispose some individuals to aortic valvular inflammation in response to normal oscillatory shear stress (73).

A contradicting role for Notch1 signalling has been observed in promoting TLR-mediated chronic inflammation in CAVD. Diseased aortic valves show increased expression of TLR2 and TLR4, and stimulation of human aortic VICs with peptidoglycan and lipopolysaccharide (LPS) resulted in increased expression of pro-osteogenic BMP2, BMP4, and RUNX2 (142). TLR4 stimulation in aortic VICs results in increased Notch1 expression, and upregulation of downstream pro-inflammatory NF-κB and ICAM-1 and pro-osteogenic BMP-2 that is partially Notch1-dependent and reproducible with the Notch1-ligand, Jagged1 (165, 166). Shear-stress induced Notch1 activation in a Piezo1-dependent fashion has previously been demonstrated in hepatic endothelial cells (167). These differing roles of the Notch1 pathway in CAVD highlight that there are likely heterogenous pathophysiological pathways leading to clinical CAVD, and suggest that the mechanosensitive Notch1 pathway may be suppressed in congenitally predisposed CAVD, while being accentuated in LPS-induced chronic inflammation.

## The Unsolved Quest for Medical Therapy of Aortic Stenosis

Risk factors for the development of CAVD overlap heavily with traditional risk factors for atherosclerotic coronary artery disease, such as male gender, smoking, hypertension, low-density lipoprotein cholesterol (LDL-C) levels and lipoprotein(a) levels (168). Unlike coronary disease, however, modification of these factors has not been shown to significantly alter the mortality associated with aortic stenosis. At present, United States and European guidelines recommend no effective medical therapy for AS beyond the control of hypertension (4, 169).

Randomised trials examining the utility of lipid-lowering using HMG-CoA reductase inhibitors have failed to show an effect on CAVD progression. The largest SEAS trial randomised 1873 patients with asymptomatic, mild-to-moderate AS to either 40 mg simvastatin plus 10 mg ezetimibe or placebo and showed a reduction in ischaemic events, but no difference in mortality or in progression to symptomatic valve disease or valve replacement over 4 years follow-up (38). Meta-analysis incorporating data from smaller trials also failed to demonstrate any benefit from statin therapy in CAVD.

Identification of abnormal angiotensin-converting enzyme (ACE) and angiotensin II (Ang II) expression in stenotic valves has implicated the renin-angiotensin system (RAS) in the pathophysiology of CAVD (170). ACE and Ang II are known to promote fibrosis and ventricular remodelling following myocardial infarction and have been implicated as a mediator of LV hypertrophy in response to pressure overload (171–173). Animal models of aortic stenosis and small-scale randomised trials have shown reverse remodelling of LV hypertrophy with RAS inhibition (127, 174), but no trial so far has demonstrated a reduction in mortality or echocardiographic progression of AS (175).

Observational studies have examined the effects of anti-osteoporotic agents on the progression of calcific aortic valve disease. Bisphosphonates are pyrophosphate analogs which inhibit osteoclast activity, thereby reducing bone resorption and turnover (176). Multiple small observational studies have observed suggested reductions in the rate of change of echocardiographic parameters of AS in patients on bisphosphonate treatment compared with matched control patients (177–180). The largest retrospective registry study to date, however, showed no significant change in AS progression in patients on bisphosphonate therapy (181). These observational studies are confounded by the comparison of patients with and without osteoporosis, a disorder of calcium homoeostasis that may promote cardiovascular calcification independent of the action of bisphosphonates (182). Denosumab is a monoclonal antibody inhibitor of RANK ligand, which mimics the activity of OPG and is used in the treatment of osteoporosis. Given the protective properties of OPG against cardiovascular calcification, denosumab has raised interest in the prevention of CAVD and has been shown to inhibit porcine aortic VIC calcification *in vitro* (183). The SALTIRE2 trial randomised 150 non-osteoporotic patients with moderate calcific AS to either denosumab, the bisphosphonate agent alendronate or placebo, and found no significant difference detected in the progression of CAVD as assessed by computerised tomography (CT) calcium scoring, doppler echocardiography and 18F-sodium fluoride positron emission tomography (184, 185).

Given the inflammatory nature of the disease process, anti-inflammatory therapies remain of interest in arresting CAVD. Randomised trials examining the role of anti-inflammatory therapies in atherosclerotic heart disease have shown some promising benefits. The CANTOS trial assessed canakinumab, a monoclonal antibody interleukin-1β (IL-1β) antagonist, in patients with previous myocardial infarction and raised inflammatory markers and showed a small reduction in rates of myocardial infarction, accompanied by an increase in deaths attributable to infections (186). Further trials of the anti-inflammatory drug colchicine have also demonstrated a reduction in major adverse cardiovascular events in patients at high risk of myocardial infarction (187, 188). Despite showing no mortality benefit, these trials demonstrate the potential for anti-inflammatory interventions to alter the progression of atherosclerotic cardiovascular disease. To date, no randomised trials have assessed the utility of anti-inflammatory interventions in the treatment of CAVD.

Balloon aortic valvuloplasty is a temporising treatment for symptomatic, severe aortic stenosis which has a limited role in the TAVI-era due to the inevitable disease recurrence and high mortality that follows (189). Histological study of excised valve leaflets in patients undergoing AVR who had previously undergone balloon valvuloplasty shows a distinct pattern of leaflet microfracture healing with collagen deposition, fibroblast proliferation and true bone formation (190, 191). The comparatively good long-term outcomes following balloon mitral valvuloplasty may suggest a role of the greater transaortic shear stress in promoting rapid restenosis following balloon valvuloplasty (192). There have been limited investigation of the potential for anti-inflammatory interventions to increase the longer-term success of balloon valvuloplasty. A non-randomised pilot trial of external beam radiation therapy following balloon valvuloplasty showed low rates of echocardiographic restenosis at 12 months (193). Experimental trial of aortic valvuloplasty using a balloon coated with the antiproliferative agent paclitaxel showed a reduction in collagen formation, cell proliferation and restenosis compared to plain balloon valvuloplasty in a rabbit model of AS, however, there is no available human data on the potential safety or efficacy of drug-coated balloon valvuloplasty (194, 195).

## Valve Replacement

Aortic valve replacement remains the only effective treatment at reducing mortality and morbidity from symptomatic aortic stenosis. In the last decade, transcatheter aortic valve replacement (TAVR) has emerged as the dominant alternative to surgical aortic valve replacement (SAVR) with low thromboembolic and bleeding complication rates and comparable survival in patients at high and intermediate surgical risk (196–198). TAVR additionally allows for valve replacement to occur with a far lesser systemic inflammatory response compared to surgical valve replacement, allowing study of the effect of valve replacement on the inflammatory milieu of CAVD (199). Increased levels of circulating inflammatory mediators associated with severe aortic stenosis have been observed to reverse with valve replacement; with significant reductions in circulating intermediate phenotype monocytes noted at 3 months post-TAVR and 6 months post-SAVR (200).

Study of monocyte functional properties before and after TAVR has shown increased levels of monocyte activation, increased monocyte-endothelial cell adhesion and increased activation of the Mac-1 complement receptor in blood monocytes collected prior to TAVR (52). A similar activation state was shown to be induced *in vitro* by exposing monocytes to a microfluidic model of aortic stenosis, directly implicating the high shear rate endured by circulating blood cells in patients with severe aortic stenosis in the perpetuation of chronic inflammation and establishing TAVR as anti-inflammatory therapy. Most interestingly, the mechanoreceptor Piezo1 has been identified as a mediator of this shear-dependent activation of monocytes (52). This identifies a potential therapeutic target, the inhibition of which might interrupt the vicious cycle of shear-stress induced inflammatory acceleration of aortic valve stenosis.

Exposing naive VICs to patient serum collected prior to and 1 month following TAVR implantation has shown that serum from patients post-TAVR have increased concentrations of IL-1β and TNFα and induced quiescence in VICs, previously activated to a myofibroblastic phenotype by pre-TAVR serum (201). Interestingly, this effect was blunted when VICs were cultured on stiffer substrates, reinforcing the importance of both reduction in shear stress and removal of the pro-inflammatory substrate in the de-escalation of chronic inflammation following TAVR.

## Working Toward Uncoupling the Vicious Cycle of Inflammation in Calcific Aortic Valve Disease

As treatment options and clinical outcomes continue to improve in coronary artery disease and heart failure, the search for therapeutic targets in valvular heart disease and CAVD must become a leading priority in cardiovascular research. Fortunately, novel techniques and approaches are breaking new ground in the effort to understand the pathophysiology of CAVD.

High-throughput sequencing technology allows for large scale evaluation of genetic variants, gene expression and quantification of protein and small molecules within a tissue sample, yielding vast amounts of data (202). The application of this multi-omics approach has implicated many novel pathways in the development of CAVD (203). High throughput omics strategies also have the ability to reveal mechanisms behind previously observed observations in CAVD. Traditional focussed research methods for example have identified lipoprotein (a) as a significant risk factor for the development of calcific AS (204, 205). Genome-wide association study (GWAS), however, have revealed a single nucleotide polymorphism with a strong independent predictive association with the development of AS; and multi-omics study has identified potential effector proteins and expressed genes linking Lp(a) to valvular calcification (206, 207). Wang et al. utilised multi-omic screening of explanted human aortic valve specimens to identify dual-specificity phosphatase 26 (DUSP26) as an upregulated gene promoting aortic valve calcification in CAVD, subsequently using *in vivo* mouse and *in vitro* human VIC studies to confirm that DUSP26 promotes CAVD via upregulation of dipeptidyl peptidase 4 (DPP4) (208). Moving forward, multi-omics studies have great potential to identify genes, proteins and small molecules that may be targetted in medical treatment for CAVD.

Accurate delineation of pathophysiological heterogeneity remains a major gap in our understanding of the pathophysiology of CAVD and AS. While clinical practice currently only distinguishes between trileaflet and bileaflet aortic valve disease, AS is a clinical syndrome that likely represents the end result of varying pathophysiological processes. Studying different points in the temporal development of CAVD is crucial to improving our understanding of the varying initiating pathways of early CAVD as well as the common pathways of established CAVD that lead to clinically significant aortic stenosis.

In addition to identifying new molecular targets, existing therapeutic agents may be repurposed in the treatment of CAVD (209). As mechanistic studies identify new mediators of CAVD, existing drugs such as DPP4 inhibitors currently used in the treatment of diabetes may become candidates for the treatment of CAVD (208). Similarly, proprotein convertase subtilisin/kexin type 9 (PCSK9) inhibitors have shown efficacy in the treatment of coronary artery disease, and upregulation of PCSK9 has been identified in human CAVD and promotes VIC calcification *in vitro* (210). A randomised trial is currently underway to examine the effect of PCSK9 inhibition on the progression of CAVD (EPISODE trial, NCT04968509).

Finally, innovative approaches to disease modelling are required for mechanistic and therapeutic research in CAVD. Given the complex relationships between VICs, substrate stiffness and shear stress conditions, complex *in vitro* models are required to accurately simulate the effect of interventions on VIC differentiation and calcification. The use of 3D hydrogels and bioreactors has allowed for simulation of the conditions experienced by aortic valve leaflets *in vivo* (211, 212). Existing animal models too are limited in their applicability to human CAVD given the reliance on hyperlipidaemic models, which may not represent the same disease process as human CAVD (213).

## Conclusion

Calcific aortic valve disease remains a common condition associated with high mortality and morbidity. Aortic stenosis can be readily diagnosed with a stethoscope and echocardiography and has a long-dormant phase, followed by a rapid acceleration of disease. This rapid progression of the severity of aortic stenosis is driven by escalating chronic inflammation of the valve tissue and thus far, no medical therapy has been established to prevent this progression.

Mechanosensitive pathways link mechanical stresses to the progression of CAVD via activation of circulating monocytes and platelets, triggering of endothelial-mesenchymal transition, upregulation of proinflammatory pathways and promotion of VIC differentiation. These observations suggest a feedback mechanism by which the increased leaflet stiffness and altered shear stress conditions of early CAVD perpetuate and accelerate inflammation and progression of valvular sclerosis and calcification. Identification and *in vivo* quantification of mechanosensing pathway activity in CAVD may show utility in prognostication and identification of patients at risk of rapid disease progression.

Finally, inhibition of mechanosensing feedback loops holds promise as a novel therapeutic target for the prevention and treatment of clinically significant aortic stenosis. It is imperative that further research is conducted to better delineate the mechanisms responsible for initiation and perpetuation of inflammation in CAVD and the mechanosensitive feedback pathways responsible which may offer diagnostic and therapeutic targets.

## Author Contributions

All authors listed have made a substantial, direct, and intellectual contribution to the work, and approved it for publication.

## Conflict of Interest

The authors declare that the research was conducted in the absence of any commercial or financial relationships that could be construed as a potential conflict of interest.

## Publisher’s Note

All claims expressed in this article are solely those of the authors and do not necessarily represent those of their affiliated organizations, or those of the publisher, the editors and the reviewers. Any product that may be evaluated in this article, or claim that may be made by its manufacturer, is not guaranteed or endorsed by the publisher.
